# Engineering microbes for tolerance to next-generation biofuels

**DOI:** 10.1186/1754-6834-4-32

**Published:** 2011-09-21

**Authors:** Mary J Dunlop

**Affiliations:** 1 University of Vermont, School of Engineering, 33 Colchester Ave, Burlington, VT 05405, USA

## Abstract

A major challenge when using microorganisms to produce bulk chemicals such as biofuels is that the production targets are often toxic to cells. Many biofuels are known to reduce cell viability through damage to the cell membrane and interference with essential physiological processes. Therefore, cells must trade off biofuel production and survival, reducing potential yields. Recently, there have been several efforts towards engineering strains for biofuel tolerance. Promising methods include engineering biofuel export systems, heat shock proteins, membrane modifications, more general stress responses, and approaches that integrate multiple tolerance strategies. In addition, *in situ *recovery methods and media supplements can help to ease the burden of end-product toxicity and may be used in combination with genetic approaches. Recent advances in systems and synthetic biology provide a framework for tolerance engineering. This review highlights recent targeted approaches towards improving microbial tolerance to next-generation biofuels with a particular emphasis on strategies that will improve production.

## Introduction

Microbes can be engineered to produce biologically-derived replacements for gasoline, diesel, and aviation fuel. Although much research has focused on ethanol as a biogasoline, there are many other biofuels that offer advantages such as high energy density, low freezing point, and compatibility with the existing fuel storage and distribution infrastructure [1-3]. Next-generation biofuels, such as long-chain alcohols, fatty-acid-derived, and isoprenoid-derived fuels offer promise as new biofuels and can be synthesized by microbes. These fuels are being developed as either supplements or drop-in replacements for existing petroleum fuels. Because there is active research on many next-generation fuels, this review highlights general tolerance strategies and discusses areas where mechanisms may only work for certain classes of fuels.

Next-generation biofuels have many advantages, but the fuels are often toxic to microorganisms. Therefore, the inherent tolerance of the host may limit production potential. Microbes that can survive in hydrocarbon-rich environments have been isolated [4,5], however these strains are rarely suitable for use as biofuel production hosts. Recent efforts have suggested that it may be possible to transfer tolerance mechanisms to a suitable production strain. The ideal host is a well studied organism with good genetic tools available that can be engineered for both biofuel production and tolerance.

It is often assumed that increasing tolerance will improve yields. There are several studies where this is the case [6-8], but also well documented examples where increases in tolerance have no effect or have even decreased yield [9-11]. Because biofuel tolerance is complex, and often intimately linked to general stress response, it can be difficult to predict the effect of a given tolerance strategy.

**Figure 1:**
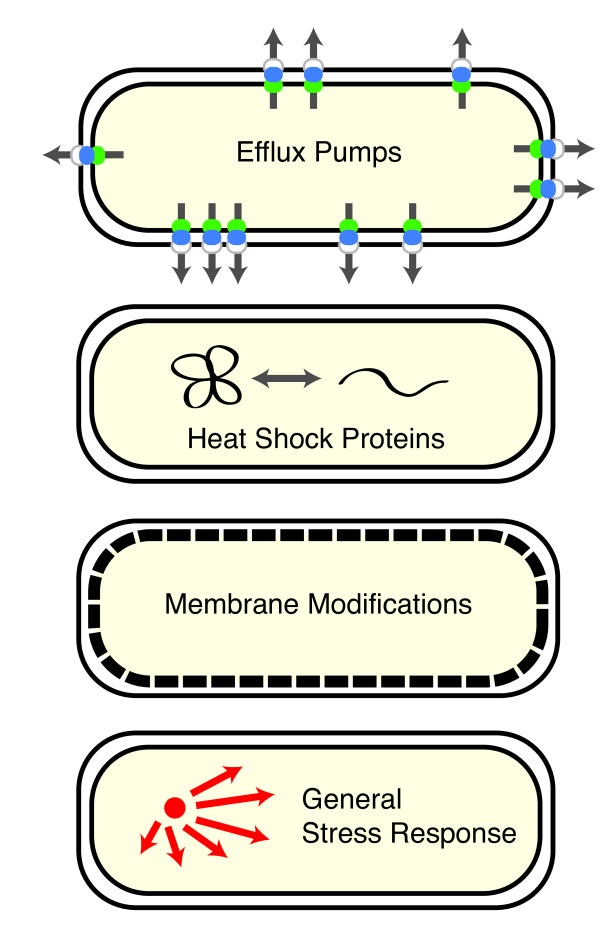
**Biofuel tolerance mechanisms**.

This review highlights recent advances in tolerance engineering for the production of next-generation biofuels. Particular emphasis is placed on targeted approaches for improving tolerance that can be applied in engineered strains. For more comprehensive coverage of topics related to stress and tolerance in microbial bioprocessing, a recent article by Nicolaou *et al. *[12] provides excellent depth. The present review first describes why biofuels are toxic to cells. Next, it surveys specific mechanisms for improving tolerance such as expression of efflux pumps, heat shock proteins, membrane modifying proteins, and activation of general stress response genes (Figure 1). It then discusses how these approaches have altered yields in biofuel production strains, exploring the link between tolerance and production. Finally, it reviews media supplementation strategies and *in situ *approaches for recovering biofuel that can be used to reduce the need for highly tolerant strains.

### Mechanisms of toxicity

Solvent stress has been studied extensively, though almost exclusively in the context of exogenous addition of the solvent rather than intracellular production. There are several comprehensive reviews on this subject [4,5,12,13], and therefore an overview of these mechanisms is provided here. The antimicrobial activity of a solvent is highly correlated with its hydrophobicity, which determines the extent to which the solvent accumulates in the cytoplasmic membrane. The accumulation of solvents in the membrane has several consequences: it increases the permeability of the membrane, diminishes energy transduction, interferes with membrane protein function, and increases fluidity. The increase in membrane permeability can allow the release of ATP, ions, phospholipids, RNA, and proteins. Direct effects include reduced ATP levels, reduced ATP synthesis, and diminished proton motive force, all of which are detrimental to energy maintenance in the cell. Solvents in the cell membrane can also negatively affect membrane protein function, further interfering with essential cellular processes such as nutrient transport. Finally, the increase in membrane fluidity changes the stability and structure of the cell membrane. In summary, solvents interfere with the membrane's ability to act as a barrier, and interrupt key processes such as transport and energy transduction.

Toxicity levels vary widely across different types of biofuels. The toxicity of a biofuel is correlated with how well it partitions in the cell membrane (this is often measured by the octanol-water partition coefficient, log P_ow_, which is a good surrogate for the harder-to-measure membrane partition coefficient) [4,5,14]. Longer chain alcohols are generally more toxic than short chain alcohols. And toxicity typically increases with solvent hydrophobicity. A summary of toxic levels of several next-generation biofuels is given in Table 1. It should be noted that some promising biofuels do not show antimicrobial activity. For example, biodiesels such as farnesene and fatty-acid derived fuels do not inhibit cell growth at moderate concentrations [15,16].

**Table 1:** Toxicity levels for next-generation biofuels

	Tolerance (% v/v)	Microorganism	Reference
**Biogasolines**			

Butanol	1.5%	*Escherichia coli*	[1]
	1.5%	*Zymomonas mobilis*	[1]
	1.5%	*Pseudomonas putida*	[17]
	1.6%	*Clostridium acetobutylicum*	[55]
	2.0%	*Saccharomyces cerevisiae*	[1]

2-Methyl-1-butanol (2MB)	0.1%	*Escherichia coli*	[70]

3-Methyl-1-butanol (3MB)	0.1%	*Escherichia coli*	[71]

Geraniol	0.05%	*Escherichia coli*	[7]

1-Propanol	5.0%	*Escherichia coli*	[28]

**Biodiesels**			

Farnesyl hexanoate	1.0%^a^	*Escherichia coli*	[7]

Geranyl acetate	0.5%^a^	*Escherichia coli*	[7]

**Bioaviation fuels**			

Pinene	0.5%^a^	*Escherichia coli*	[7]

Limonene	0.025%	*Escherichia coli*	[7]

^a^Biofuel is inhibitory, but not lethal; percentages listed are where growth drops to 75% of the normal, unstressed levels.

Tolerance properties can also vary across different species and strains. For example, growth of most microbes is inhibited by 1% to 2% (v/v) butanol, though there are examples of adapted strains of *Pseudomonas *that can tolerate up to 6% (v/v) [17]. The values in Table 1 represent intrinsic tolerance levels for common host strains. Many efforts have identified strains with increased tolerance, and approaches relevant for biofuel production are discussed here. However, it is important to recognize that what works for one biofuel may not work for another, and the host strain may only support a subset of the tolerance engineering strategies.

### Efflux pumps

Efflux pumps are membrane transporters that recognize and export toxic compounds from the cell using the proton motive force. Physiologically, they play an important role in cell survival by exporting a wide range of substrates, including bile salts, antimicrobial drugs, and solvents [18-20]. Although best studied for their role in antibiotic resistance, there are several pumps that appear to be specific for solvents. For example, the solvent resistance pump (*srpABC*) from *Pseudomonas putida S12 *has been shown to export hexane, octanol, and several other hydrocarbons [21]. *P. putida *DOT-T1E harbors three solvent resistant efflux pumps, collectively known as the toluene tolerance genes (*ttg*) [22]. In addition to these solvent-specific efflux pumps, many broad-range efflux pumps can also export hydrocarbons. For example, the *acrAB*-*tolC *pump from *Escherichia coli *provides tolerance to hexane, heptane, octane, and nonane [23]. Three *mex *pumps from *Pseudomonas aeruginosa *have also been shown to export solvents [24]. The best-studied solvent tolerance pumps are those from the resistance-nodulation-division (RND) family in Gram-negative bacteria, but many other organisms also harbor efflux pumps [20].

RND efflux pumps in Gram-negative bacteria are composed of three protein subunits that span the inner and outer membranes: the inner membrane protein responsible for substrate recognition and proton antiport, the periplasmic linker that acts as a bridge between the inner membrane pump, and finally the outer membrane channel. All three subunits are essential for pump function.

Recent experiments by the author and collaborators have demonstrated that heterologously expressed RND efflux pumps can improve tolerance to biofuels [7]. Efflux pump operons were cloned from a broad range of organisms and expressed individually in an *E. coli *host. Improvements in tolerance were observed when cells were grown in the presence of biogasoline, biodiesel, and bioaviation fuel. Importantly, pump expression was also shown to improve biofuel production.

Expression of efflux pumps is a promising engineering strategy for many biofuels, however there is mounting evidence that these pumps are not effective at exporting short-chain alcohols. Although several studies have shown that these alcohols can induce expression of pumps [25-27], recent work has consistently demonstrated that the pumps do not increase tolerance. Ankarloo *et al. *showed that *E. coli acrAB *does not improve tolerance to ethanol or 1-propanol [28]. Furthermore, when expression of *acrAB *was indirectly induced by the addition of salicylate, cells were actually less tolerant to the alcohols. A similar conclusion was suggested by two independent studies that identified *acrAB *deletions among those that increased isobutanol tolerance [9,29]. Finally, none of the members of a library of heterologously expressed efflux pumps were able to increase *E. coli *tolerance to *n*-butanol or 3-methyl-1-butanol [7]. These recent studies strongly suggest that efflux pumps are not effective at exporting short-chain alcohols, and may even reduce tolerance in some instances.

For longer chain alcohols, alkanes, alkenes, and cyclic hydrocarbons, efflux pumps offer a promising strategy for increasing biofuel tolerance and production. Pumps that have been shown to be effective at improving biofuel tolerance are listed in Table 2. There are several avenues for future research in this area. Directed evolution may help produce designer pumps that are specific to particular biofuels or effective at providing tolerance to other toxins such as inhibitors from deconstructed biomass. In addition, it may be necessary to tune efflux pump expression, as it is well known that overexpression of membrane proteins can be detrimental [30,31]. Initial studies have demonstrated that efflux pumps can be used to improve biofuel tolerance and yield; future work will optimize their functionality in a production environment.

**Table 2:** Efflux pumps that exhibit biofuel tolerance

Pump^a^	Bacterial species	Reference
AcrB	*Escherichia coli*	[72]

AcrF	*Escherichia coli*	[73]

MexF	*Pseudomonas putida*	[7]

NP_745594	*Pseudomonas putida*	[7]

SrpB	*Pseudomonas putida*	[25]

TtgB	*Pseudomonas putida*	[22]

TtgE	*Pseudomonas putida*	[22]

TtgH	*Pseudomonas putida*	[22]

MexB	*Pseudomonas aeruginosa*	[24]

MexD	*Pseudomonas aeruginosa*	[24]

MexF	*Pseudomonas aeruginosa*	[24]

NP_250708	*Pseudomonas aeruginosa*	[7]

MexF	*Azotobacter vinelandii*	[7]

YP_960752	*Marinobacter aquaeolei*	[7]

YP_957870	*Marinobacter aquaeolei*	[7]

YP_692684	*Alcanivorax borkumensis*	[7]

AcrB	*Pseudoalteromonas haloplanktis*	[7]

^a^Protein name or GenBank accession number for inner membrane protein

### Heat shock proteins

Stress from short-chain alcohols has many parallels with heat shock [32]. Heat shock proteins are molecular chaperones that are involved in the synthesis, transport, folding, and degradation of proteins. Under stress, they prevent protein aggregation and assist with refolding; under normal conditions, they play a housekeeping role. Genomic studies have repeatedly revealed heat shock proteins among those that are upregulated in response to solvent stress and there is evidence that they may be good engineering targets for improving biofuel tolerance and yield.

Several recent studies have shown consistent upregulation of heat shock proteins in response to solvent stress. Brynildsen *et al. *subjected *E. coli *to isobutanol stress and measured changes in the transcriptional response network, finding strong activation of RpoH, a heat shock sigma factor [33]. Rutherford *et al. *conducted a similar study to measure transcriptional changes in *E. coli *under *n*-butanol stress and found several genes related to heat shock and protein misfolding, including *rpoH*, *dnaJ*, *htpG*, and *ibpAB *[27]. In a study subjecting *Clostridium acetobutylicum *to exogenous butanol stress, Tomas *et al. *showed that many of the known heat shock proteins were overexpressed (*groESL*, *dnaKJ*, *hsp18*, *hsp90*) [34]. Evolved isobutanol-tolerant strains of *E. coli *also showed modifications to the chaperone GroL [29]. Similar findings were obtained in a comprehensive study by Alsaker *et al. *examining butanol, butyrate, and acetate stress in *C. acetobutylicum *[35]. Stress from all three metabolites upregulated expression of *dnaK*, *groES*, *groEL*, *hsp90*, *hsp18*, and several other stress genes. It is clear that expression of heat shock proteins is altered in response to solvent stress.

Biofuel tolerance can be improved by overexpressing heat shock proteins. Tomas *et al. *showed that overexpression of GroESL improved tolerance in *C. acetobutylicum *[8]. Importantly, these tolerance improvements also led to an increase in butanol yields. Other studies overexpressing heat shock proteins in *Lactobacillus plantarum *[36] and *E. coli *[37] provide corroborating evidence for the ability of heat shock proteins to increase biofuel tolerance.

### Membrane modifications

Solvent-tolerant microbes can actively change their membrane composition to block entry of solvents. In non-tolerant strains, the presence of solvents disrupts the cell membrane structure and has a profound impact on physiological function, ultimately leading to cell death [4,13]. To counter this effect, tolerant strains can shift the composition of the fatty acids in their membrane to block the entry of solvents. A well studied example of this phenomenon is the shift from *cis *to *trans *unsaturated fatty acids, which can be catalyzed by the *cis*-*trans *isomerase (*cti*) [38,39]. An increase in the ratio of *trans *to *cis *fatty acids is correlated with a decrease in membrane fluidity and a corresponding increase in solvent tolerance [40]. As a longer-term response, cells can alter the ratio of saturated to unsaturated fatty acids to exclude solvents or stabilize the membrane [4]. In addition, modifications to phospholipid headgroups or phospholipid chain length have been shown to increase solvent tolerance [5].

Membrane modifications that decrease permeability may not be effective independent strategies for improving biofuel production since they could trap fuel molecules within the cell, however they may be useful in concert with other mechanisms. Individually, membrane modifications are likely to increase tolerance to exogenous solvents, but there is evidence that production will not be improved [10]. A combined strategy that utilizes both membrane modifications and other approaches mirrors natural examples from solvent-tolerant microbes and may prove to be an effective engineering approach. For example, membrane modifications may be helpful in combination with export pumps. Alternatively, the timing and rate of fatty-acid biosynthesis could be regulated in response to production levels. The ability to optimize membrane composition will lead to increases in tolerance, but the relationship between tolerance and production must be carefully considered with this approach.

### Other stress responses and potential for systems engineering

Recent studies have explored the solvent stress response with the twin goals of characterizing biofuel stress and identifying strategies for combating it. Consistent themes from these studies indicate that respiration, general stress response mechanisms, and membrane proteins are altered in response to biofuel [12]. These may serve as engineering targets, either for improving yield or by use as switches to activate expression of other tolerance mechanisms.

Brynildsen *et al. *[33] identified 16 transcription factors in *E. coli *to be significantly perturbed by isobutanol stress. A key finding from the study was that respiration is highly altered under isobutanol stress. Rutherford *et al. *[27] identified genes related to energy production and conversion, amino acid transport and metabolism, and signal transduction as the mostly highly affected by *n*-butanol stress in *E. coli*. A major finding of their work was that reactive oxygen species were highly elevated during *n*-butanol stress; they suggest that genes that alleviate oxidative stress may be valuable engineering targets. A study by Reyes *et al. *[37] used a genomic enrichment library to find genes from *E. coli *that enhanced *n*-butanol tolerance. Similar to previous studies, membrane and stress response proteins were shown to play an important role in *n*-butanol tolerance. The biggest improvements came from overexpression of *entC *and *feoA*, which are involved in iron transport and metabolism. In addition, the authors identified gene deletions that improved tolerance, including acid resistance-related *astE *and inner membrane protein *ygiH*. A similar genomic library approach was used to search for *n*-butanol tolerance genes in *C. acetobutylicum *[41]. Several genes were identified, including a transcription factor that is hypothesized to regulate transitional-phase events, *CAC1869*, which provided an 81% improvement in growth relative to the control strain under butanol stress. Minty *et al. *evolved isobutanol tolerant strains of *E. coli *and found several genes related to the cell envelope and stress response in the tolerant strains [29]. Their findings suggest that post-transcriptional regulatory proteins may play an important role in tolerance. In addition, attenuation of the stress response by the global regulator RpoS improved tolerance, a surprising finding given that non-adapted strains upregulate *rpoS *expression [29,33]. This apparent inconsistency may be due to the interaction between RpoS with mutations to *hfq *and *acrAB *in the adapted strains. Although the studies discussed above differ in their use of *n*-butanol versus isobutanol, tests comparing the two indicate that responses are similar [9,33]. General themes that emerge from the butanol response network studies are that respiration is clearly affected by the stress, membrane protein expression is disproportionally altered, and general stress response mechanisms are activated.

The stress response mechanisms identified in these studies may also play a key role in future systems engineering efforts. Detailed understanding of the response to biofuel stress and the genes that are implicated in these changes may be useful in engineering cellular control systems [42]. For example, expression of solvent-responsive proteins such as ArcA, Fur, and PhoB, which are activated indirectly by isobutanol, could be used to turn on a switch that indicates that the cell is experiencing isobutanol stress [33]. This switch could be used to control expression of production pathway genes, or export mechanisms that would serve to alleviate the stress [31]. Similar strategies have been used with great success to control expression of other metabolically engineered production pathways [43].

Although the main focus of this review is on targeted tolerance mechanisms, traditional strain engineering will also play a crucial role in improving tolerance. Classical strain improvement methods such as chemostat-mediated adaptation, mutagenesis, and evolutionary engineering are valuable approaches when the desired phenotype involves complex genotypic solutions [44]. These approaches have proved successful for engineering environmental tolerance [45], and can be used in combination with genome shuffling or targeted genomic approaches [46]. In addition, recent advances in recombineering methodologies introduce powerful tools for rapid introduction of mutations and subsequent analysis [47-49].

### Synergistic approaches to tolerance

Solvent tolerant microbes employ multiple resistance mechanisms. For example, a comparative genomic study on efflux pumps in *P. putida *strains demonstrated that the degree of solvent tolerance is highly correlated with the number efflux pumps the strain harbors; strains with several different types of pumps were more likely to be highly tolerant to solvents [50]. Furthermore, an individual strain may utilize diverse resistance strategies. *P. putida *DOT-T1E uses three efflux pumps, a *cis*-*trans *isomerase, and can form vesicles to isolate the toxic solvents [5]. Microbes that metabolize hydrocarbons generally have additional tolerance mechanisms such as the ability to degrade the toxins, cell wall strengthening isomerases, genes specific to biofilm formation at an oil-water interface, and the ability to produce biosurfactants [51,52]. Based on the natural examples of solvent-tolerant microbes it is likely that integrating multiple strategies will greatly improve biofuel tolerance of a production host.

Genome-level studies on biofuel tolerant strains have consistently found that it is necessary to alter the expression of multiple genes to provide the greatest benefit [9,27,33,53]. For example, simultaneous disruption of five unrelated genes provided significant improvements to isobutanol tolerance in a study by Atsumi *et al. *[9]. No single mutation was solely responsible for the improvements in tolerance, and all five were needed to reproduce the levels observed in evolved strains. Recent work by Goodarzi *et al. *showed that combined computational and experimental approaches may help to improve tolerance [54]. The authors experimentally determined the effect of single-gene perturbations and then used these data in a computational model to accurately predict the effects of combining multiple perturbations. Such hybrid approaches will be very powerful for the creation of biofuel tolerant strains.

### Link between tolerance and production

Increased biofuel tolerance appears to be necessary, but not sufficient for increased fuel production. Several studies show improvements in tolerance that correspond to clear increases in biofuel yield. For example, ethanol production in an engineered strain of *Saccharomyces cerevisiae *was improved 15% when its ethanol and glucose tolerance were improved through global transcriptional machinery engineering [6]. With the tolerance improvements, production levels reached 98% of the theoretical yield. *C. acetobutylicum *can produce butanol and acetone, but butanol toxicity is a major inhibitor of solvent production [55]. By overexpressing the *groESL *heat shock genes, Tomas *et al. *reduced butanol toxicity in *C. acetobutylicum *by 85%, which resulted in 40% improvements in butanol production [8]. Limonene tolerance in *E. coli *was improved by heterologously expressing an efflux pump and the corresponding strain showed a 64% improvement in limonene yield [7]. End-product toxicity is a known problem in industrial-scale production of chemicals by microorganisms. For example, *E. coli *has been used as an industrial host to produce the bulk chemicals 1,4-butanediol and 1,3-propanediol. In both cases engineering devoted to improving tolerance was required to make production cost effective [56,57]. Thus, there is clear evidence that tolerance improvements can increase production.

However, improvements in tolerance are not sufficient to guarantee an increase in biofuel yields. *C. acetobutylicum *studies have shown that butanol tolerance can be increased by genetic changes that modify the fatty-acid composition of the membrane, but these effects inhibited solvent production [10,11]. A study by Atsumi *et al. *isolated an isobutanol tolerant strain of *E. coli *through a serial transfer experiment [9]. Although the strain was more tolerant to exogenous isobutanol, the production yields were identical to those of the original engineered production strain. Because biofuel production pathways may disrupt the metabolic network of the cell it is difficult to predict how changes in a particular gene product will alter biofuel production. In particular, tolerance improvements that result from the initiation of stress response pathways may have broad-ranging effects, which preclude a clear-cut link between tolerance and yield.

It is worth noting that production yields can exceed tolerance levels. Toxicity levels measured using exogenous addition of biofuel correspond to levels that inhibit growth, but production often continues long after growth stops, allowing yields to exceed native tolerance levels [58,59]. Despite this, biofuel toxicity still limits production and it will be necessary to improve tolerance to further increase yields.

### Recovery technologies and media supplements

In addition to genetic manipulations, advances in technologies for removing biofuel from the bioreactor or buffering its impacts through media supplementation can help ease the burden of toxicity. Much of the work on extraction methods has been developed for the purpose of improving fermentation for ethanol or butanol production. Key technologies for *in situ *recovery and their benefits and drawbacks have been reviewed elsewhere [55,60]; approaches include gas stripping, liquid-liquid extraction, pervaporation, and perstraction. Gas stripping bubbles a gas such as N_2_, CO_2_, or H_2 _through the reaction volume, which captures the solvents. The solvents can then be separated and collected using a condenser and the gas reused. A recent paper showed a doubling in yields when butanol was continuously extracted from the fermentation broth by gas stripping [61]. Liquid-liquid extraction is similar to gas stripping, but uses a water-insoluble solvent to capture the biofuel. Pervaporation and perstraction use a selective membrane to remove solvents from the bioreactor.

Many candidates for next-generation biofuels are insoluble in water. This property may simplify recovery strategies, however the lack of solubility does not imply that the biofuel is not toxic. Indeed, several hydrocarbons have been shown to have very poor solubility, but still exhibit significant antimicrobial properties [13]. In addition, in some cases compounds produced intracellularly may become trapped inside the cell, requiring further energy input for extraction [1].

The addition of compatible solutes to the growth medium can also help to mitigate the effects of biofuel toxicity [12]. For example, trehalose, proline and other amino acids have been shown to protect yeast against ethanol stress [62,63]. Supplementation with protectants such as betaine or inositol may also help improve biofuel tolerance [1,12,62]. Overcoming toxicity limitations will likely require a combination of genetic manipulations, advances in bioreactor recovery methods, and optimization of growth conditions.

## Conclusions

As metabolic engineering continues to improve biofuel production, yields will reach levels that are toxic to cells. This end-product inhibition places a limit on the amount of biofuel that can be produced. Approaches for increasing tolerance to biofuels have been the focus of several recent studies that characterize the stress response caused by biofuels, and develop strategies for increasing tolerance with the goal of increasing yield.

In addition to the end product, other inhibitors from the biofuel production process may also need to be addressed through tolerance engineering. For example, when biomass is deconstructed, residual products such as cellulosic hydrolysate can exhibit cytotoxicity [64-66]. Several reviews on growth inhibition by ligocellulosic hydrolysate have been published recently, which provide insight into the mechanisms of toxicity [67-69]. The tolerance strategies discussed here may also be effective for countering these inhibitors.

There are several key conclusions that can be drawn from recent studies on tolerance engineering for next-generation biofuels: first, tolerance mechanisms are likely to be specific for particular classes of biofuel. For example, RND efflux pumps are effective at exporting several biodiesels and bioaviation fuels, but are less promising for short-chain alcohols. Second, in addition to exploring native tolerance strategies through the use of genomic libraries, adaptation, and mutagenesis studies, it is also worth studying expression of heterologous genes. Third, multiple tolerance mechanisms are likely to be necessary and may have synergistic effects. Several studies on tolerance have shown that epistatic interactions between genes are necessary to improve tolerance. Fourth, tolerance improvements do not necessarily correlate with increases in yield. Although it is necessary to improve tolerance to increase yield, simply increasing tolerance may not increase yield. Finally, optimized growth conditions and recovery strategies that are integrated with the biofuel production infrastructure will help ease the burden caused by end-product toxicity. Combined approaches that use strain engineering with effective recovery technologies will likely be necessary.

Future tolerance engineering efforts should be conducted in biofuel production strains when possible so that improvements in production yields can be measured in conjunction with tolerance. Researchers should explore expression of heterologous genes in addition to modifications to the native genome. Other microorganisms, especially those that are naturally adapted to hydrocarbon-rich environments, may be valuable sources of tolerance mechanisms even if they are not ideal production hosts. In some cases there may be obvious targets for metabolic engineering, but methods for heterologous library construction should also be explored. These new approaches should be used in combination with native tolerance strategies, discovered through classical strain engineering, genome shuffling, or genomic library approaches. In particular, methods that support the exploration of synergistic approaches to tolerance will be especially valuable. Tolerance engineering will be essential as production of next-generation biofuels continues to improve.

## Competing interests

MJD is a coauthor on a patent application on the topic of 'Modified host cells with efflux pumps' (application number 13/115,925).
